# Investigation of Factors Related to the Week 1 Cumulated Ambulation Score in Patients With Proximal Femoral Fractures Post-surgery Using Decision Tree Analysis

**DOI:** 10.7759/cureus.55407

**Published:** 2024-03-02

**Authors:** Ryotaro Hiramatsu, Shohei Minata, Shinsuke Imaoka

**Affiliations:** 1 Rehabilitation, Oita Oka Hospital, Oita, JPN

**Keywords:** decision tree analysis, orthopaedic surgery, rehabilitation, cumulated ambulation score, proximal femoral fracture

## Abstract

This study aimed to identify factors associated with the Cumulated Ambulation Score (CAS) in the first week post-surgery (Week 1 CAS) in patients with proximal femoral fractures. Proximal femoral fractures are prevalent in the elderly, with rising incidence rates and significant social and functional implications. The ability to walk postoperatively is a critical determinant of patient prognosis.

The study included 53 patients out of 79 who underwent surgery for proximal femoral fractures at the orthopedics department of Oita Oka Hospital from January 2021 to December 2021. Exclusion criteria were pre-existing walking difficulties, inability to be evaluated in the first postoperative week, non-weight bearing post-surgery, and complications during hospitalization. The physical therapy program followed Oita Oka Hospital's clinical path, starting ambulation with a walker within the first week post-surgery. Data collected included patient background, surgical techniques, pre-injury ambulatory status, and pre-admission residential environment. Physical function assessments one week postoperatively included range of motion (ROM), manual muscle testing (MMT), pain evaluation (NRS), and CAS. Statistical analyses involved the Shapiro-Wilk test, independent t-test, Mann-Whitney U test, chi-square test, and decision tree analysis using classification and regression trees (CART).

Patients were categorized into 'favorable' and 'poor' groups based on Week 1 CAS. Significant differences in dementia presence and pre-admission living environment were noted between groups. Knee extension MMT at Week 1 postoperatively showed a significant difference. The decision tree analysis identified knee extension MMT as the primary variable, with a threshold of 3.5. In patients with MMT below 3.5, dementia presence was a secondary factor, with 81% in the poor CAS group. In patients with MMT above 3.5, knee extension strength continued to be significant. The model's accuracy was 64%, with precision at 71%, recall at 63%, and an F1-score of 0.67.

The study highlights knee extension MMT and dementia presence as significant factors influencing Week 1 CAS in patients with proximal femoral fractures. The poor CAS group had a higher proportion of dementia and weaker knee extension MMT.

Dementia hinders rehabilitation effectiveness, impacting recovery in knee extension strength and CAS. The decision tree analysis provided an intuitive understanding of variable interrelationships, emphasizing knee extension strength as the primary factor, followed by dementia in cases with lower MMT scores.

This study elucidated factors related to Week 1 CAS in postoperative patients with proximal femoral fractures. Knee extension MMT emerged as the initial factor, followed by the presence of dementia, influencing Week 1 CAS. These findings are crucial for rehabilitation planning and long-term prognostic predictions in this patient population. However, the study's limitations include potential selection bias and a small sample size, necessitating further research for enhanced predictive accuracy.

## Introduction

Proximal femoral fractures have a high incidence rate among the elderly, and the number of cases has been rising steadily, with a predicted continued increase in the future [1]. It is reported that approximately 20% of patients with proximal femoral fractures may become bedridden a year after injury due to the time required to recover walking ability [2]. Therefore, proximal femoral fractures not only have a significant social impact due to their prevalence but also from the perspectives of functional and life prognoses [3].

The prognosis of patients with proximal femoral fractures is reported to be dependent on their walking ability [4], making the assessment of walking capacity essential for predicting outcomes. Factors related to functional prognosis include age, pre-injury walking ability, and dementia [5], with additional reports suggesting that walking ability in the first week post-surgery is also related to prognosis [6]. Hence, evaluating walking ability in the first week after surgery is considered important not only for planning discharge destinations and setting goals with post-discharge life in mind but also for long-term prognostic predictions.

In recent years, there has been an increase in reports concerning the Cumulated Ambulation Score (CAS) as an assessment of early postoperative mobility in patients with proximal femoral fractures [7,8]. The CAS, proposed by Foss et al. [9], is an evaluation score that quantifies the ability of patients with proximal femoral fractures in terms of bed mobility, transfer ability, and walking ability across six levels. This evaluation score was translated into Japanese in 2020, and its reliability and validity have been reported [10]. According to Mashimo et al. [11], the CAS evaluated on the seventh day after surgery, referred to as 'Week 1 CAS,' is an indicator for predicting discharge to home, with a reported cutoff value of 4 points. Thus, a postoperative one-week CAS of 4 or more points may be a predictor of discharge home. However, while there are reports on the correlation between CAS and prognosis, it is not clear what physical functions or conditions are associated with one-week postoperative CAS. Therefore, investigating functional factors related to CAS, which in turn explores mobility-related functional factors, is believed to be beneficial in providing effective physical therapy.

Therefore, investigating function related to the one-week postoperative CAS is to identify the one-week postoperative indicators associated with discharge home. We believe that identifying this will help in beneficial rehabilitation in the early postoperative period.

## Materials and methods

Participants

The participants were 53 cases out of 79 patients who underwent surgery for proximal femoral fractures (femoral neck fractures, intertrochanteric femoral fractures) diagnosed at the orthopedic department of Oita Oka Hospital from January 2021 to December 2021, excluding those who met the exclusion criteria. The exclusion criteria were as follows: (1) those who had difficulty walking upon hospital admission; as for the criteria for gait difficulty, we excluded cases in which the patient has difficulty walking with a walking aid, (2) those who could not be evaluated during the first postoperative week due to severe cognitive decline or complications that occurred during hospitalization, and (3) those who were prescribed a period of non-weight bearing after surgery.

The physical therapy program for patients after proximal femoral fracture surgery was based on the clinical path of our hospital; getting out of bed started on the day after surgery, and walker-assisted walking began in the first week post-surgery (Table 1).

**Table 1:** Postoperative Physical Therapy Program

	Clinical Pathway
Preoperative	Preoperative Information Collection and Training of the Healthy Side's Muscle Strength
Postoperative Day 1	Transfer from Bed to Wheelchair
Postoperative Day 7	Initiation of Walker-Assisted Ambulation ※Subject to early transition upon physician's approval
After Postoperative Day 7	Discharge or Transfer to Another Hospitals

Methods

The design of this investigation was an observational cross-sectional study. Data were collated retrospectively from medical records.

Information Pertaining to Patient Background Factors

Variables related to patient background factors included age, gender, presence of cognitive impairment, fracture type, surgical technique, ambulatory status prior to injury, and pre-admission residential environment. Surgical techniques were categorized into two modalities: arthroplasty and osteosynthesis. The ambulatory status prior to injury was classified into three categories: ambulating independently, using a cane, and utilizing a walker. The residential status prior to hospital admission was dichotomized into living at home or residing in a facility.

Information on Physical Function at One Week Post-Surgery

The parameters concerning physical function one week postoperatively included the following: the range of motion (ROM) for hip flexion, hip extension, hip adduction, hip abduction, knee flexion, and knee extension; manual muscle testing (MMT) for hip flexion, hip abduction, knee extension, and ankle plantar flexion; assessment using the Numerical Rating Scale (NRS); and the CAS. These evaluations were conducted by the responsible therapist on the day of the assessment, at one week post-surgery.

For the Week 1 postoperative evaluation, the ROM was measured using a goniometer at 5-degree intervals, following the methodology described by Lea et al [12]. MMT was performed with attention to compensatory movements, in accordance with the techniques outlined by Mendell et al [13]. The CAS involved scoring three basic activities at one week postoperatively: (1) bed transfers, (2) sitting down and standing up from a chair or an armrest-equipped wheelchair, and (3) indoor walking (utilization of walking aids permitted). These activities were scored as follows: independent - 2 points, with assistance - 1, impossible - 0 points, resulting in a maximum score of six points. Subsequently, a Week 1 CAS of 4 points or higher was classified as the 'favorable group' (indicating good progress), while a score of 3 points or lower was defined as the 'poor group' (indicating poor progress).

Statistical analyses

Statistical processing commenced with the execution of the Shapiro-Wilk test to ascertain the normality of the data before conducting each test. Subsequently, for the comparison between the two groups based on the CAS at one week postoperatively, continuous variables were analyzed using the independent t-test, ordinal variables were examined using the Mann-Whitney U test, and nominal variables were evaluated with the chi-square test.

Next, the CAS at one week postoperatively (divided into the favorable and poor groups) was treated as the dependent variable. Univariate analysis was conducted to identify significant independent variables, which were then used in decision tree analysis. The algorithm employed for the decision tree analysis was the classification and regression trees (CART), with a maximum depth set at 2. Statistical analyses were performed using Python 3.9.13, and the significance level was set at 5%.

## Results

**Figure 1:**
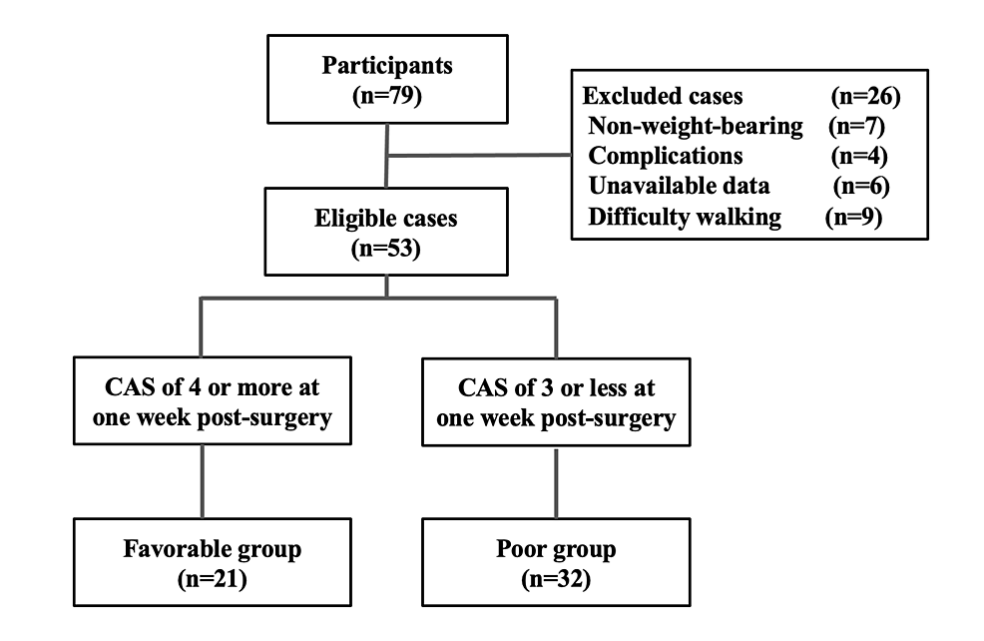
Subject Flowchart CAS: cumulated ambulation score

Upon classifying the subjects into two groups (favorable and poor) based on their Week 1 CAS, 21 individuals were categorized as the favorable group, and 32 as the poor group (Figure 1).

The results of comparing the Week 1 CAS (favorable and poor groups) are presented in Table 2.

**Table 2:** Comparisons Based on Cumulated Ambulation Score (CAS) at One Week Post-Surgery Values are presented as mean ± standard deviation (minimum-maximum) BHA: Bipolar hip arthroplasty, ROM: range of motion, MMT: manual muscle test, NRS: numerical rating scale

Parameters	Favorable group (n=21)	Poor group (n=32)	P-value
Age	84.2±7.9	86.7±6.2	0.231
Gender (male/female）	3/18	9/23	0.323
Dementia (yes/no）	7/14	24/8	0.006
Fracture type (femoral neck/ trochanteric）	11/10	14/18	0.361
Operation (BHA/ osteosynthesis）	15/17	10/12	0.738
Pre-injury walking pattern (Independent/ T-cane/walker）	16/3/2	19/6/7	0.400
Pre-hospitalization living environment (home/ institutional setting）	20/1	20/12	0.017
ROM			
Hip flexion	89.8±8.0	85.8±11.0	0.134
Hip extension	-2.8±6.5	-8.6±11.3	0.052
Hip adduction	7.4±4.1	8.5±3.5	0.288
Hip abduction	21.2±6.3	18.5±9.4	0.306
Knee flexion	114.5±13.9	106.7±18.2	0.090
Knee extension	-6.5±6.3	-5.7±7.0	0.687
MMT			
Hip flexion (1/2/3)	0/15/6	2/24/5	0.174
Hip abduction (1/2/3)	1/17/3	3/27/0	0.070
Knee extension (2/3/4/5)	6/8/4/3	21/8/2/1	0.005
Ankle flexion (2/3)	19/1	28/0	0.254
NRS	4.7±1.6	4.3±2.7	0.417

Among the background factors, significant differences were noted in the presence of dementia and the pre-admission living environment. In terms of physical function at Week 1 post-surgery, a significant difference was observed in the knee extension MMT (p<0.05).

**Figure 2:**
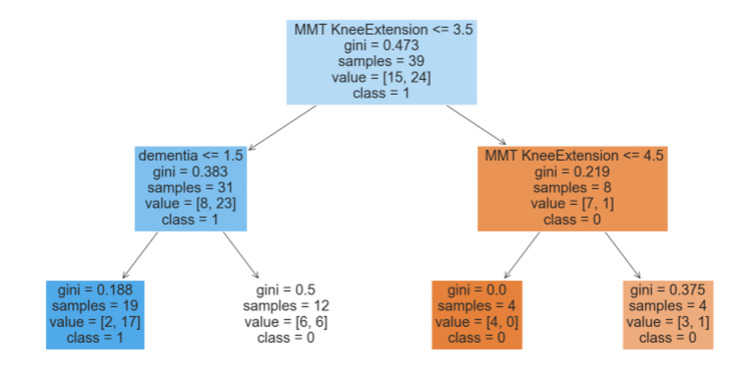
Decision Tree Analysis MMT knee extension: manual muscle test knee extension

The decision tree generated by CART analysis is displayed in Figure 2.

**Figure 3:**
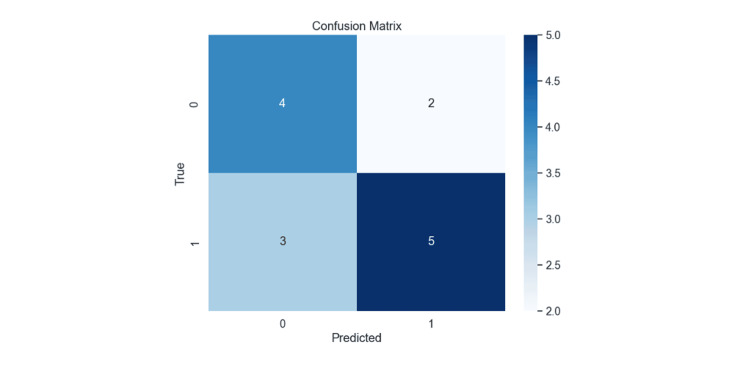
Confusion Matrix of the Model

In this model, knee extension MMT was selected as the criterion at the first node, dividing the subjects into two groups at a threshold of 3.5. In the group with knee extension MMT less than 3.5, the presence of dementia was chosen at the second node, where 81% of those with dementia fell into the poor Week 1 CAS group. Among those without dementia, 50% were classified into the poor Week 1 CAS group. In the group with knee extension MMT greater than or equal to 3.5, knee extension MMT was again selected at the second node. All individuals with knee extension MMT greater than or equal to 4.5 fell into the favorable Week 1 CAS group. Even with a knee extension MMT of less than 4.5, 68% were categorized in the favorable Week 1 CAS group. The accuracy of the generated model was 64%, with a precision of 71%, a recall of 63%, and an F1-score of 0.67 (Figure 3).

## Discussion

This study aimed to elucidate factors related to the Week 1 CAS, an indicator predictive of discharge to home, in patients with proximal femoral fractures. Given the impact of age and dementia on treatment prioritization in cases of proximal femoral fractures [14,15], this study utilized decision tree analysis to progressively determine factors associated with Week 1 CAS. As a result, knee extension MMT and the presence of dementia emerged as significant factors related to Week 1 CAS. Specifically, a knee extension MMT score below 3.5 and the presence of dementia were found to be associated with poorer Week 1 CAS outcomes.

Comparing the favorable and poor Week 1 CAS groups, it was evident that the poor group had a higher proportion of dementia and more individuals living in facilities before admission, alongside weaker knee extension MMT. Previous research [16] has also demonstrated the influence of dementia and physical function on walking ability postoperatively. Therefore, our study suggests that dementia, pre-admission living environment, and knee extension MMT are potentially related to Week 1 CAS.

In the decision tree analysis, knee extension MMT emerged as the primary variable at the first node. Postoperative patients with proximal femoral fractures exhibit a significant reduction in knee extension strength on the affected side [17], leading to decreased walking ability [18] and increased fall risk [19]. This study found that weaker knee extension strength tends to correlate with poorer Week 1 CAS outcomes. Consequently, knee extension MMT appears to significantly influence Week 1 CAS.

At the second node, dementia status was identified as a key variable for those with knee extension MMT scores below 3.5. In cases where patients had dementia and a score below 3.5, 81% fell into the poor Week 1 CAS category. It has been reported that dementia can hinder improvements in CAS due to challenges [20] in providing appropriate rehabilitation. Thus, the presence of dementia may delay recovery in knee extension strength, leading to lower Week 1 CAS scores. In groups with knee extension MMT scores above 3.5, knee extension MMT continued to be a significant factor [21], underscoring the strong association between knee extension strength and Week 1 CAS.

With regard to the decision tree analysis performed in this study, items that were significantly different in the univariate analysis were put in as explanatory variables, following the method of Lucca et al [22].Therefore, decision tree analysis used in this study, characterized by its intuitive tree structure and hierarchical arrangement of factors, facilitated the understanding of the interrelationships among variables [23]. Our results indicate that knee extension MMT is the primary factor influencing Week 1 CAS, followed by the presence of dementia in cases where knee extension MMT is below 3.5 [24].

The decision tree model achieved an accuracy of 64%, a precision of 71%, a recall of 63%, and an F1-score of 0.67. These metrics suggest that the model had relatively few misclassifications, making it potentially useful for clinical decision-making. However, this study has limitations, including potential selection bias due to being conducted in a single facility and a small sample size. Moreover, while some improvement in model accuracy might be expected with a larger sample size, further data collection and longitudinal studies are necessary to enhance the model's predictive accuracy. There is also a need to consider analytical methods, such as how to conduct univariate analysis and how to select variables, taking into account the characteristics of the data set. In addition, since there are insufficient assessment items in this study, there is a need to increase the number of assessment items and to study the issue again.

## Conclusions

In this study, our objective was to progressively elucidate factors related to the Week 1 CAS in patients postoperatively treated for proximal femoral fractures. The findings revealed that initially, knee extension MMT was a factor influencing the postoperative Week 1 CAS, followed by the presence or absence of dementia.
